# Multiple Intravenous Administrations of Human Umbilical Cord Blood Cells Benefit in a Mouse Model of ALS

**DOI:** 10.1371/journal.pone.0031254

**Published:** 2012-02-03

**Authors:** Svitlana Garbuzova-Davis, Maria C. O. Rodrigues, Santhia Mirtyl, Shanna Turner, Shazia Mitha, Jasmine Sodhi, Subatha Suthakaran, David J. Eve, Cyndy D. Sanberg, Nicole Kuzmin-Nichols, Paul R. Sanberg

**Affiliations:** 1 Center of Excellence for Aging and Brain Repair, University of South Florida, College of Medicine, Tampa, Florida, United States of America; 2 Department of Neurosurgery and Brain Repair, University of South Florida, College of Medicine, Tampa, Florida, United States of America; 3 Department of Molecular Pharmacology and Physiology, University of South Florida, College of Medicine, Tampa, Florida, United States of America; 4 Department of Pathology and Cell Biology, University of South Florida, College of Medicine, Tampa, Florida, United States of America; 5 Department of Psychiatry, University of South Florida, College of Medicine, Tampa, Florida, United States of America; 6 Saneron CCEL Therapeutics, Inc., Tampa, Florida, United States of America; 7 Ribeirão Preto School of Medicine, University of São Paulo, São Paulo, Brazil; University of Nebraska Medical center, United States of America

## Abstract

**Background:**

A promising therapeutic strategy for amyotrophic lateral sclerosis (ALS) is the use of cell-based therapies that can protect motor neurons and thereby retard disease progression. We recently showed that a single large dose (25×10^6^ cells) of mononuclear cells from human umbilical cord blood (MNC hUCB) administered intravenously to pre-symptomatic G93A SOD1 mice is optimal in delaying disease progression and increasing lifespan. However, this single high cell dose is impractical for clinical use. The aim of the present pre-clinical translation study was therefore to evaluate the effects of multiple low dose systemic injections of MNC hUCB cell into G93A SOD1 mice at different disease stages.

**Methodology/Principal Findings:**

Mice received weekly intravenous injections of MNC hUCB or media. Symptomatic mice received 10^6^ or 2.5×10^6^ cells from 13 weeks of age. A third, pre-symptomatic, group received 10^6^ cells from 9 weeks of age. Control groups were media-injected G93A and mice carrying the normal hSOD1 gene. Motor function tests and various assays determined cell effects. Administered cell distribution, motor neuron counts, and glial cell densities were analyzed in mouse spinal cords. Results showed that mice receiving 10^6^ cells pre-symptomatically or 2.5×10^6^ cells symptomatically significantly delayed functional deterioration, increased lifespan and had higher motor neuron counts than media mice. Astrocytes and microglia were significantly reduced in all cell-treated groups.

**Conclusions/Significance:**

These results demonstrate that multiple injections of MNC hUCB cells, even beginning at the symptomatic disease stage, could benefit disease outcomes by protecting motor neurons from inflammatory effectors. This multiple cell infusion approach may promote future clinical studies.

## Introduction

Amyotrophic lateral sclerosis (ALS) is a neurodegenerative disorder characterized by a loss of upper and lower motor neurons. Symptoms include spasticity, fasciculations, muscle weakness and atrophy, combined with progressive paralysis ultimately leading to death, usually within three to five years of diagnosis. The sporadic form of ALS (sALS) predominates, with only 5–10% of cases identified as genetically linked; of those that have a familial etiology (fALS), 20% show missense mutations in the Cu/Zn superoxide dismutase (SOD1) gene on chromosome 21 [1]. In sALS cases, the etiology of the disease is still undefined. However, the clinical presentation and underlying pathology of sALS and fALS are similar. Although numerous hypotheses about the etiopathology of this multifactorial disease have been proposed [2]–[4] including neurovascular pathology [5], reliable treatment to halt disease progression and restore function remains elusive.

Cell therapy may be a promising treatment for ALS. Although motor neuron replacement is possible, this treatment strategy should take into account the multifocal motor neuron degeneration and death [6]. The roles of cell-based therapeutics might be more practical “as modifiers of the ALS-specific microenvironment” [7] or serving to “detoxify the local environment around dying motor neurons” [8], therefore providing protection for motor neurons and retarding disease progression. Neuroinflammation, comprised mainly of astrocyte and microglial activation, is a central feature in ALS, and directly contributes to neuronal death [9]–[11]. Therefore, attempting to modulate inflammation, combined with other neuroprotective strategies in ALS, seems a more realistic approach than neuronal replacement [12], thus eliminating the need for neural cell sources.

Numerous reports demonstrate the functional multipotency of non-neural cells such as bone marrow, peripheral blood and umbilical cord blood cells [13]–[16]. Based on the recently proposed concept of biofunctional multipotency of stem cells to mediate systemic homeostasis, stem cell multipotency should be considered in planning for therapeutic applications [17]. In an ALS clinical trial, autologous ex vivo expanded mesenchymal cells from bone marrow were transplanted directly into the thoracic spinal cord of patients [18], [19]. While beneficial effects were described only in a few patients, no overall changes in disease progression were noted. A second report [20] confirmed the lack of changes in neurological progression of sALS patients transplanted intravenously with allogenic peripheral blood CD34+ hematopoietic stem cells, however, some transplanted cells were found in motor neuron sites of the spinal cord. Likely, the cell sources chosen, specifically bone marrow and peripheral blood, may not have been the optimal choices.

Human umbilical cord blood (hUCB) cells may be preferable to other potential cell sources [21]–[25]. The hUCB cells are low in pathogenicity and are immunologically immature. Hematopoietic progenitors from cord blood are rich in the most primitive stem cells [26]–[31] and are capable of developing into cells of various tissue lineages including neural cells [32]–[34]. Additionally, cord blood lymphocytes express cytokine receptor profiles (interleukins [IL]-2, IL-4, IL-6, IL-7, tumor necrosis factor [TNF]-α, and interferon-γ) at lower levels than adult blood cells [35] and produce great amounts of the anti-inflammatory cytokine IL-10 [36]. Moreover, umbilical cord blood cells secrete trophic factors, which can directly support neuronal survival [37].

In recent years, reports have shown that hematopoietic umbilical cord blood cells are versatile instruments for the treatment of various disorders including neurodegenerative diseases [21], [23], [24]. The mononuclear cell fraction derived from human umbilical cord blood (MNC hUCB) has been effective in the treatment of experimental stroke [38]–[40], traumatic brain injury [41], spinal cord injury [42] and Alzheimer's disease [43]. It was also shown that intravenous (iv) administration of MNC hUCB into aged rats demonstrably improved the microenvironment of the aged brain [44]. Using the G93A SOD1 mouse model of ALS, we have previously demonstrated [45] that a single systemic iv administration of MNC hUCB cells, at the low dose of 10^6^ cells, delayed disease progression by at least 2–3 weeks and modestly increased lifespan. More recently, we investigated the optimal MNC hUCB cell dosage, verifying that a larger dose of 25×10^6^ cells administered intravenously into pre-symptomatic G93A mice delayed disease onset by 15% and significantly increased lifespan by 20–25% [46]. The effects were likely due to enduring inhibition of various inflammatory effectors, inhibition that promoted motor neuron survival. However, converting this large mouse dosage into a single human equivalent dose [47] would require approximately 20 units of cord blood, impractical in a clinical setting. A more feasible approach would be delivery of multiple smaller cell doses during disease progression, thus providing ongoing protection for motor neurons.

The aim of this study was to determine the effect of systemic multiple MNC hUCB cell administrations into pre-symptomatic and symptomatic G93A SOD1 mice modeling ALS. Importantly, this is the first time that a preclinical translational study has been designed to address efficacy of a proposed cell treatment at the symptomatic stage of the disease.

## Results

Of the total 108 G93A SOD1 mice used in the study, seven mice (*Group1* – one, *Group 2* – two, *Group 3* – three, *Group 4* – one) were excluded due to death precipitated by conditions other than disease progression, more specifically, anesthetic complications during cell or media administrations. The number of injections per group was: *Group 1* (*Gr 1*, 2.5×10^6^ MNC hUCB, symptomatic) - 6.50±0.27 (range 4–8), *Group 2* (*Gr 2*, 1×10^6^ MNC hUCB, symptomatic) - 6.00±0.23 (range 4–8), *Group 3* (*Gr 3*, 1×10^6^ MNC hUCB, pre-symptomatic) - 11.65±0.36 (range 9–15), and *Group 4* (*Gr 4*, Media-injected, symptomatic) - 5.47±0.23 (range 4–7). Although the range of injection numbers was similar between *Gr 1* and *Gr 2*, the number of mice receiving 8 injections at symptomatic stage in *Gr 1* was n = 5 and in *Gr 2* was n = 2. The total number of injected cells was: *Gr 1* - 16.25±0.67×10^6^, *Gr 2* - 6.00±0.23×10^6^, and *Gr 3* - 11.60±0.37×10^6^ cells.

Before distributing mice into groups, the ΔCT, proportional to the expression of mutant SOD1 gene, was determined. Results showed similar ΔCT for all G93A mice and no significant differences between groups: *Gr 1* - 5.96±0.07, *Gr 2* - 5.92±0.05, *Gr 3* - 5.91±0.05, *Gr 4* - 6.05±0.33 cycles.

### Effect of multiple administrations of MNC hUCB cells on disease progression

**Figure 1:**
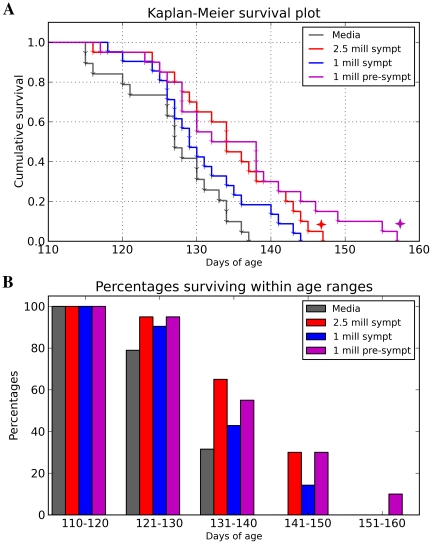
Effect of multiple MNC hUCB cell administrations on lifespan of G93A mice. (**A**) Kaplan-Meier survival curves for G93A mice receiving 2.5×10^6^ (*Gr 1*) or 1×10^6^ (*Gr 2*) cells at symptomatic disease stage and 1×10^6^ (*Gr 3*) cells pre-symptomatically. Control group was Media-injected mice (*Gr 4*). Significant (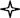) increases in survival were determined in mice receiving 1×10^6^ cells at pre-symptomatic stage (p = 0.0015) and 2.5×10^6^ cells at symptomatic stage (p = 0.0022) vs. the Media-injected group. Survival of the mouse group receiving 1×10^6^ cells pre-symptomatically tended towards significance compared to survival of mice receiving same cell dose at symptomatic stage (p = 0.0595). (**B**) Percentages of surviving mice within age ranges. Media-injected animals survived no longer than 19.5 weeks, whereas 30% of mice receiving 2.5×10^6^ (*Gr 1*, symptomatic) or 1×10^6^ cells (*Gr 3*, pre-symptomatic) and 14.3% mice administered with 1×10^6^ cells (*Gr 2*) at symptomatic stage survived more than 140 days and 10% of mice from *Gr 3* (1×10^6^ cells, pre-symptomatic) were alive for more than 150 days.

The MNC hUCB cells were intravenously administered weekly into G93A mice beginning at either pre-symptomatic (9 weeks old) or symptomatic disease stage (13 weeks old). Symptomatic mice received one of two different cell doses. Significant increases in survival were determined in mice receiving 1×10^6^ cells at pre-symptomatic stage (p = 0.0015, χ^2^ = 10.07) and 2.5×10^6^ cells at symptomatic stage (p = 0.0022, χ^2^ = 9.393) vs. the Media injected group (Figure 1A). Average lifespan of MNC hUCB administered mice was: 2.5×10^6^ (*Gr 1*, symptomatic) - 135.00±1.86 days, 1×10^6^ (*Gr 2*, symptomatic) - 130.71±1.60 days, 1×10^6^ (*Gr 3*, pre-symptomatic) - 135.60±2.47 days compared to Media-injected mice (125.58±1.40 days). Media-injected animals survived no longer than 19.5 weeks, whereas 30% of mice receiving 2.5×10^6^ (*Gr 1*, symptomatic) or 1×10^6^ (*Gr 3*, pre-symptomatic) and 14.3% mice administered with 1×10^6^ cells (*Gr 2*) at symptomatic stage survived more than 140 days and 10% of mice from *Gr 3* (1×10^6^ cells, pre-symptomatic) were alive up to 160 days (Figure 1B).

**Figure 2:**
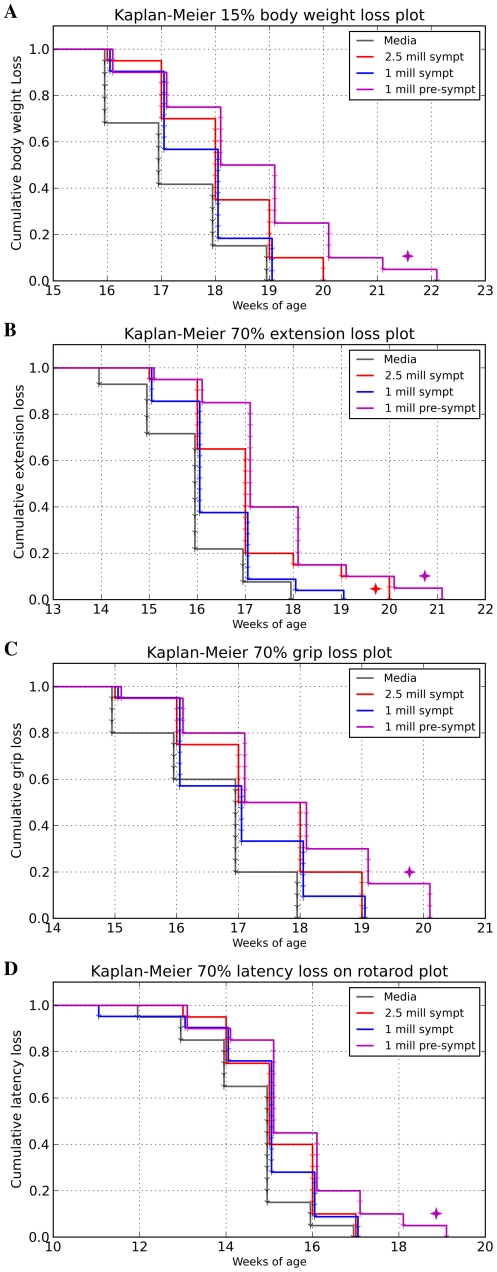
Evaluations of disease progression in G93A mice through Kaplan-Meier analysis. (**A**) Time elapsed until animals lost 15% of their maximum body weight. Mice receiving 1×10^6^ cells pre-symptomatically (*Gr 3*) significantly (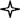) maintained body weight vs. Media (*Gr 4*) mice. A similar trend was observed in mice treated with 2.5×10^6^ cells (*Gr 1*) beginning at symptomatic disease stage. (**B**) Time elapsed until hindlimb extension scores deteriorated by 70% of the initial score. The *Gr 1* and *Gr 3* mice significantly (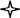) delayed decline of hindlimb extension compared to *Gr 4* mice. A significant difference was also detected between *Gr 3* and *Gr 2* mice receiving 1×10^6^ cells at pre-symptomatic or symptomatic stage of disease, respectively. (**C**) Time elapsed until muscle strength decreased by 70% from the maximum value. Mice from *Gr 3* significantly (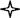) delayed muscle strength losses vs. *Gr 4*. *Gr 1* mice tended to maintain muscle strength post-transplant. (**D**) Time elapsed until rotarod latency decreased by 70% of the maximum value. Only mice from *Gr 3* performed better on the rotarod than other cell-treated mice and tended towards significance (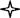) taking more time to decrease latency by over 70% of the maximum value compared to *Gr 4*.

Body weight is not only a general indicator of mouse health, but is also a valuable marker for detecting progression of muscle atrophy, and was measured weekly. As expected, body weight started to slowly decline at the symptomatic age of approximately 13–14 weeks in all G93A mouse groups. By 16 weeks of age, more than 20% of Media mice had lost 15% of their initial body weight. Although the mean body weight loss from initial measurement to the day of sacrifice for all G93A mice was 17.19±0.80%, treated animals lost weight more slowly, as they survived longer than the Media group. A Kaplan-Meier plot (Figure 2A) was constructed based on the threshold of 15% of body weight loss, a point closely corresponding to the end-stage of disease. Mice receiving 1×10^6^ cells at pre-symptomatic stage (*Gr 3*) maintained their body weight significantly longer (p = 0.0152, χ*2* = 5.894) than Media-injected mice (*Gr 4*).

Cell treated mice, in *Gr 1* (2.5×10^6^ cells, symptomatic) and *Gr 3* (1×10^6^ cells, pre-symptomatic), also displayed superior performance in other tests of functional ability. Deteriorating extension reflex was noted in G93A mice, beginning at 13 weeks of age, with extension progressively declining until the end-stage of disease. However, hindlimb extension of mice from *Gr 1* and *Gr 3* deteriorated more slowly than Media-injected mice (*Gr 4*) and mice receiving 1×10^6^ cells at symptomatic stage (*Gr 2*). At 17 weeks of age, *Gr 1* and *Gr 3* mice presented, respectively, 41.5% and 51.04% of the initial hindlimb extension scores, while mice from *Gr 2* and *Gr 4* presented 31.0% and 26.47% of initial values. The mean loss in extension scores, from the initial score until time of sacrifice, was 89.21±2.07% for all G93A mice. Similarly to the body weight analysis, Kaplan-Meier analysis was used to compare the number of weeks until extension reflex scores of mice from each group dropped more than 70% (Figure 2B). The *Gr 1* (2.5×10^6^ cells, symptomatic) and *Gr 3* (1×10^6^ cells, pre-symptomatic) mice presented significantly delayed deterioration of hindlimb extension compared to Media (*Gr 4*) mice (*Gr 1 vs. Gr 4*, p = 0.0493, χ*2* = 3.865; *Gr 3* vs. *Gr 4*, p = 0.0069, χ*2 = 7.301*). A significant difference (p = 0.0269, χ*2* = 4.895) was also detected between *Gr 3* and *Gr 2* mice receiving 1×10^6^ cells at the pre-symptomatic or symptomatic stage of disease, respectively.

In the grip strength test, G93A mice started to show decreased muscle strength at approximately 13 weeks of age, with strength progressively declining during the course of disease. The mean loss in grip strength, from maximum to end-stage, was 87.0±1.12% for all G93A mice. Kaplan-Meier analysis showed that *Gr 3* mice administered weekly with 1×10^6^ cells beginning at the pre-symptomatic stage significantly (p = 0.0358, χ*2* = 4.405) delayed loss in muscle strength vs. Media (*Gr 4*) animals (Figure 2C).

Although declines in performance on the rotarod test were observed in all mice starting at week 13, mice beginning cell treatment at the pre-symptomatic stage (*Gr 3*) demonstrated longer latency at this time. At 17 weeks of age, these mice (*Gr 3*) maintained 19.8% of initial rotarod latency, while mice from *Gr 1* (2.5×10^6^ cells, symptomatic), *Gr 2* (1×10^6^ cells, symptomatic), and Gr 4 (Media) presented 11.29%, 11.96% and 11.05%, respectively. The mean loss in rotarod latency, from a maximum value of 180 seconds to end-stage value was 99.7±0.08%, for all G93A mice. In the Kaplan-Meier analysis, only mice from *Gr 3* performed better on the rotarod test than other cell-treated mice and took longer to lose over 70% of maximum latency than the Media (*Gr 4*) group (Figure 2D).

Analysis of disease onset in mice beginning cell treatment at the pre-symptomatic stage (*Gr 3*) was performed using the Kaplan-Meier method based on a threshold of 5% of body weight loss and 15% loss in functional tests (extension reflex, grip strength, and rotarod tests). Results demonstrated that these mice at 10–14 weeks of age significantly maintained their body weight (p = 0.0355, χ*2* = 4.420) and hindlimb extension (p = 0.0142, χ*2* = 6.008) compared to media-injected animals (*Gr 4*). Muscle strength (grip test) and rotarod performance did not significantly differ between *Gr* 3 and *Gr* 4 mice.

### Immunohistochemical analysis of administered MNC hUCB cells *in vivo*

**Figure 3:**
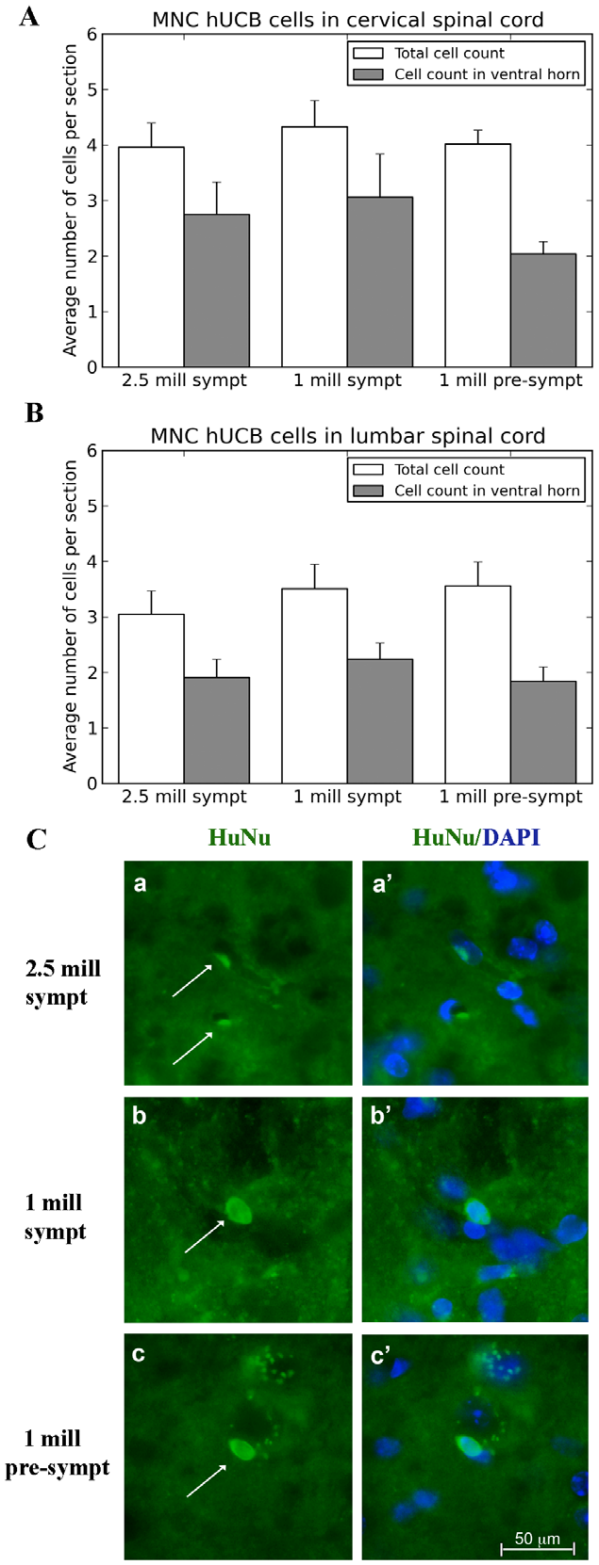
Distribution of MNC hUCB cells in the spinal cord of G93A mice. Administered MNC hUCB cells were identified immunohistochemically by a human-specific marker (HuNu) in the spinal cord of cell-treated mice at 17 weeks of age, 4 weeks (symptomatic) or 8 weeks (pre-symptomatic) post-transplant. In the total area of cervical (**A**) and lumbar (**B**) cervical spinal cord, HuNu positive MNC hUCB cells were found irrespective (p>0.05) of injected cell doses or time beginning treatment. In all cell-treated mice, more than 50% of the observed cells were in ventral horn gray matter. (**C**) Immunohistochemical staining of MNC hUCB cells in the lumbar spinal cord. MNC hUCB cells positive for HuNu (green, arrow) were detected in the lumbar spinal cord of mice receiving 2.5×10^6^ (**a**) or 1×10^6^ (**b**) cells symptomatically or 1×10^6^ cells pre-symptomatically (**c**). Cells were frequently observed inside the capillary lumen, but also in the spinal cord parenchyma. (**a′**), (**b′**), and (**c′**) are merged images with DAPI. Scale bar: a–c′ is 50 µm.

**Figure 4:**
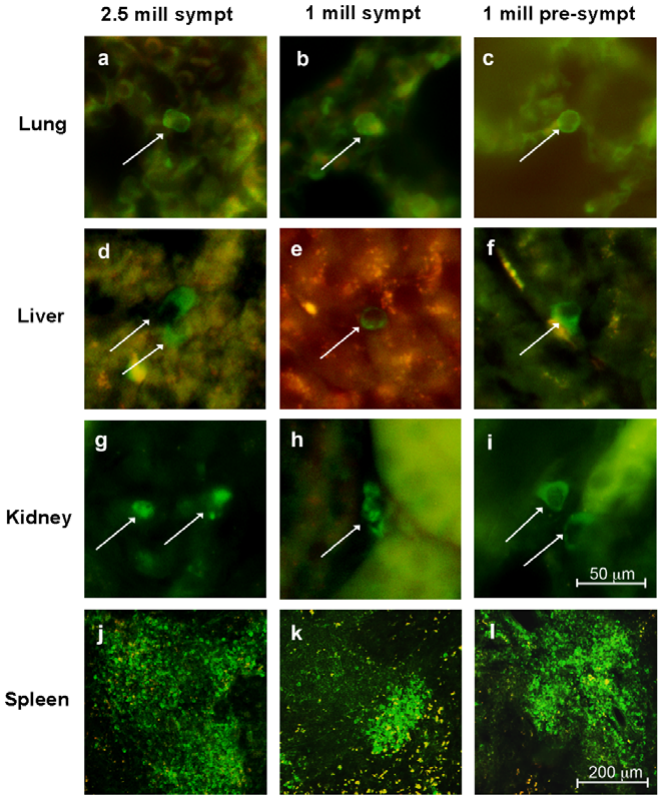
Distribution of MNC hUCB cells in the lung, liver, kidney and spleen of G93A mice. MNC hUCB cells immunohistochemically positive for HuNu (green, arrows) were detected in the lung, liver, kidney, and spleen of mice receiving 2.5×10^6^ (**a, d, g, j**) or 1×10^6^ (**b, e, h, k**) cells symptomatically or 1×10^6^ cells pre-symptomatically (**c, f, i, l**). In the liver, lung and kidney, few cells were identified. In the spleen, a high density of HuNu cells was determined in all cell-treated mice (**j–l**). Scale bar: a–i is 50 µm; j–l is 200 µm.

Administered MNC hUCB cells were identified immunohistochemically by a human-specific marker (HuNu) in the spinal cord, brain, and various abdominal organs of cell-treated mice at 17 weeks of age, 4 weeks (symptomatic) or 8 weeks (pre-symptomatic) post-transplant. Cells were widely distributed within and outside the CNS. In the cervical (Figure 3A) and lumbar (Figure 3B) cervical spinal cord, HuNu positive MNC hUCB cells were found irrespective of injected cell doses or timing of initial treatment. However, in all cell-treated mice, more than 50% of the cells were observed within the ventral horn gray matter, areas in the spinal cord known to be affected by ALS. Cells were frequently observed inside the capillary lumen, but also in the spinal cord parenchyma (Figure 3C). Some cells established in the brain, mostly in the cerebral cortex, olfactory bulb, and brainstem. In the liver, lungs and kidneys, a few cells were identified (Figure 4), but in the spleen, a high density of MNC hUCB cells was detected. Qualitative evaluation of the spleens from cell-treated animals (Figure 4, j–l) showed higher concentrations of cells in mice from *Gr 3* (1×10^6^ cells, pre-symptomatic) and *Gr 1* (2.5×10^6^ cells, symptomatic) than in *Gr 2* (1×10^6^ cells, symptomatic).

### Effect of multiple administrations of MNC hUCB cells on motor neuron survival

**Figure 5:**
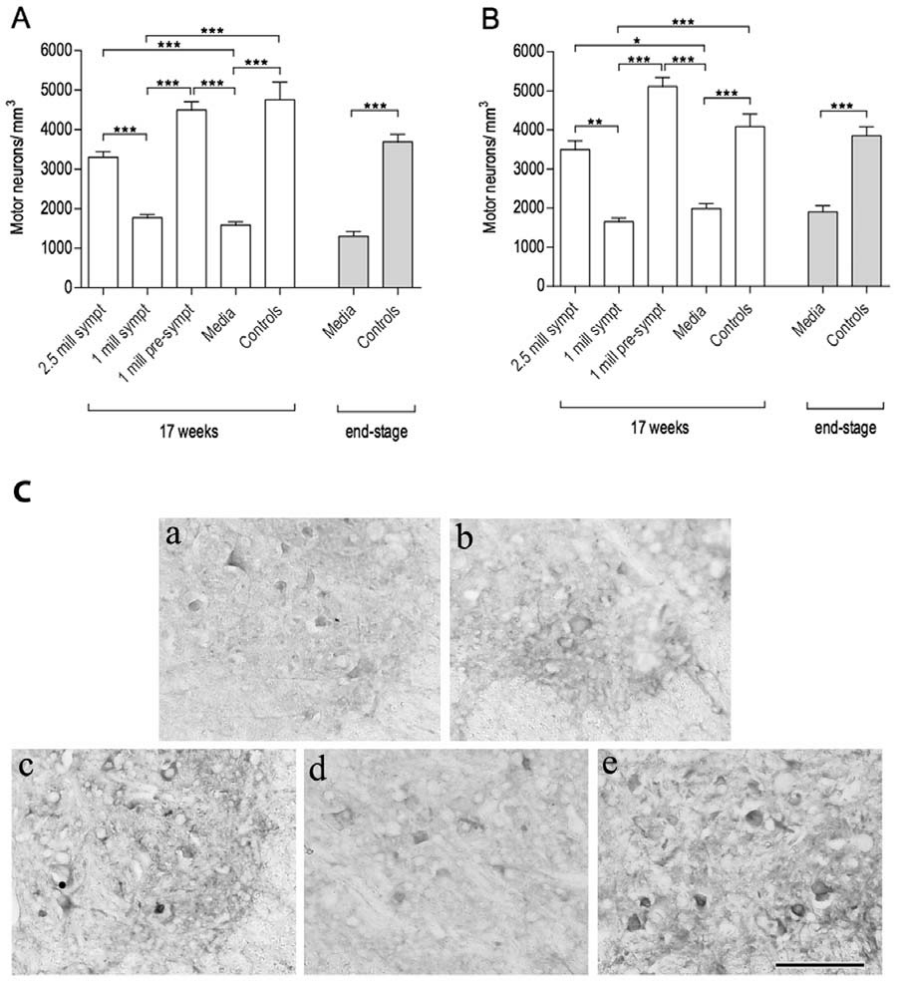
Characteristics of motor neuron survival in the spinal cord of G93A mice. Motor neuron counts were performed in the cervical (**A**) and lumbar (**B**) ventral horns of G93A mice at 17 weeks of age and at end-stage of disease. Mice receiving 2.5×10^6^ cells symptomatically (*Gr 1*) or 1×10^6^ cells pre-symptomatically (*Gr 3*) had significantly higher motor neuron densities than the Media group (*Gr 4*) at 17 weeks of age or at end-stage of disease. In both cervical and lumbar spinal cords, motor neuron densities between *Gr 2* (1×10^6^ cells, symptomatic) and Media-injected (*Gr 4*) mice showed no significant differences (p>0.05). *p<0.05, **p<0.01, ***p<0.001. (**C**) Immunohistochemical staining of motor neurons in the lumbar spinal cord of G93A mice at 17 weeks of age. Motor neuron staining for anti-choline acetyltransferase (anti-ChAT) antibody showed healthy motor neurons in controls (**a**) although only a few neurons survived in the Media-treated animals (**b**). Cell-treated mice with (**c**) 2.5×10^6^ cells symptomatically (*Gr 1*) and (**e**) 1×10^6^ cells pre-symptomatically (*Gr 3*) demonstrated higher motor neuron survival than (**d**) mice receiving 1×10^6^ cells symptomatically (*Gr 2*). Scale bar: a–e is 50 µm.

In both cervical and lumbar spinal cords, motor neuron counts were directly proportional to the survival and functional improvement observed in the mice. Figure 5 shows the results of motor neuron counts in the ventral horns of mice at 17 weeks of age and end-stage of disease. In the cervical spinal cord (Figure 5A), *Gr 1* (2.5×10^6^ cells, symptomatic) and *Gr 3* (1×10^6^ cells, pre-symptomatic) mice presented significantly higher motor neuron densities compared to Media-injected (*Gr 4*) (*G1* vs. *G4*, p<0.05; *G3* vs. *G4*, p<0.001) and *Gr 2* (1×10^6^ cells, symptomatic) mice (*G1* vs. G2, p<0.01; *G3 vs. G2*, p<0.001) groups. Motor neuron density in the cervical spinal cord of mice at 17 weeks of age was: *Gr 1* – 3,498±223.80, *Gr 2* – 1,654±92.43, *Gr 3* – 5,111±230.91, *Gr 4* - 1,989±129.20, and *Gr 5* – 4,087±321.00 number of motor neurons/mm^3^. In the lumbar spinal cord, results were similar: *Gr 3* (1×10^6^ cells, pre-symptomatic) and *Gr 1* (2.5×10^6^ cells, symptomatic) mice had significantly higher (p<0.001) motor neuron densities than the Media group (*Gr 4*) at 17 weeks of age or end-stage of disease (Figure 5B). Motor neuron density in the lumbar spinal cord of mice at 17 weeks of age was: *Gr 1* – 3,304±140.50, *Gr 2* – 1,772±86.92, *Gr 3* – 4,497±208.80, *Gr 4* - 1,589±77.22, and *Gr 5* – 4,757±444.70 number of motor neurons/mm^3^. In both cervical and lumbar spinal cords, densities from *Gr 2* (1×10^6^ cells, symptomatic) and Media-injected (*Gr 4*) mice did not differ (p>0.05) from each other, and motor neuron densities in mice from *Gr 3* and *Gr 1* were also similar (p>0.05) to controls (*Gr 5*) of same age. Figure 5C demonstrates superior survival of choline acetyltransferase (ChAT) positive motor neurons in the ventral horns of lumbar spinal cords in cell-treated animals compared to Media mice.

### Effect of multiple administrations of MNC hUCB cells on microglia and astrocytes

**Figure 6:**
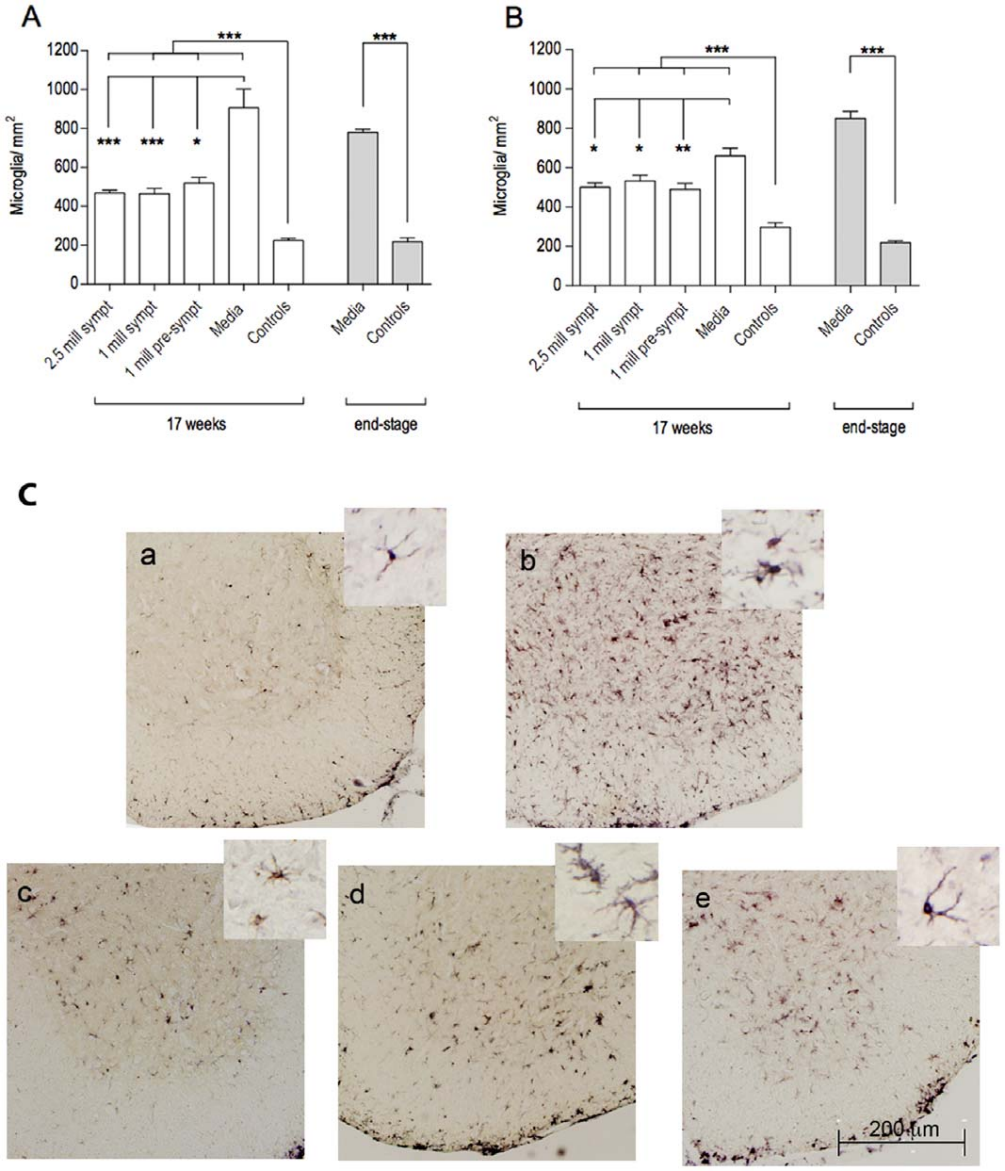
Characteristics of microglial cells in the spinal cords of G93A mice. Microglial densities were measured in the cervical (**A**) and lumbar (**B**) ventral horns of G93A mice at 17 weeks of age and at end-stage of disease. Microglial densities were significantly (p<0.001) higher in Media-injected mice (*Gr 4*) compared to control mice (*Gr 5*) of the same ages. MNC hUCB cell administrations significantly (p<0.001) decreased the number of microglia in the spinal cord of G93A mice compared to Media mice. No significant differences were detected between the cell-treated groups. *p<0.05, **p<0.01, ***p<0.001. (**C**) Immunohistochemical staining of microglia in the lumbar spinal cord at 17 weeks of age. Microglial cells stained for anti-Iba-1 antibody were sparse in controls (**a**) and microgliosis was noted in Media-treated animals (**b**). MNC hUCB cells decreased microglial density in mice from *Gr 1* (**c**), *Gr 2* (**d**), and *Gr 3* (**e**). Morphological analysis of microglial cells determined numerous activated cells with large cell bodies and short processes in Media-injected mice, whereas ramified microglia were mostly observed in cell-treated animals, particularly in *Gr 1* and *Gr 3* mice and controls (inserts in a–e). Scale bar: a–e is 200 µm; in a–e inserts is 25 µm.

At 17 weeks of age, microglial cell counts were higher in the Media group (*Gr 4*) animals, while control (*Gr 5*) mice presented the lowest densities. MNC hUCB cell administrations significantly (p<0.001) decreased the number of microglia in the cervical (Figure 6A) and lumbar (Figure 6B) ventral horns of G93A mice, although no statistical difference was detected between the cell-treated groups. Morphological analysis of microglial cells demonstrated a high number of activated cells with large cell bodies and short processes in Media-injected mice, whereas ramified microglia were mostly observed in the cell-treated animals, specifically in *Gr 1* and *Gr 3* (Figure 6C).

**Figure 7:**
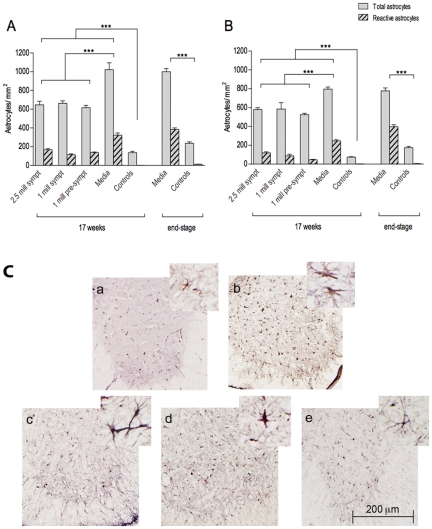
Characteristics of astrocytes in the spinal cord of G93A mice. Astrocyte densities were measured in the cervical (**A**) and lumbar (**B**) ventral horns of G93A mice at 17 weeks of age and at end-stage of disease. Astrocytic densities were significantly (p<0.001) higher in Media-injected mice (*Gr 4*) at 17 weeks of age and at end-stage of disease vs. controls (*Gr 5*) of the same ages. Significant (p<0.001) decrease in the number of astrocytes was determined in cell-treated G93A mice compared to Media mice. No significant statistical differences were detected between the cell-treated groups. Higher number of reactive astrocytes was observed in Media-injected mice at 17 weeks of age and at end-stage disease compared to cell-treated animals. ***p<0.001. (**C**) Immunohistochemical staining of astrocytes in the lumbar spinal cord of G93A mice at 17 weeks of age. Anti-GFAP antibody staining showed low astrocyte density in controls (**a**) and astrocytosis in Media-treated animals (**b**). Considerably decreased actrocytic density was observed in mice from *Gr 1* (**c**), *Gr 2* (**d**), and *Gr 3* (**e**). Astrocyte cell reactivity was also reduced in cell-treated mice vs. Media-injected mice (inserts in a–e). Scale bar: a–e is 200 µm; in a–e inserts is 25 µm.

Astrocyte cell density showed a similar pattern as the microglia in mice at 17 weeks of age. Media-injected animals (*Gr 4*) presented the highest densities, while the lowest values were noted in controls (*Gr 5*). Groups that received treatment with MNC hUCB cells presented a significant (p<0.001) decrease in astrocytic densities in the cervical (Figure 7A) and lumbar (Figure 7B) spinal cords compared to Media, with no significant difference between cell-treated groups. When the number of reactive astrocytes was assessed in each mouse group, higher proportions of these cells were observed in Media-injected mice at 17 weeks of age and end-stage disease compared to cell-treated animals. These cells were distinguished by their morphology, as exemplified in Figure 7C. The selective cell count once more demonstrated that Media-injected mice presented higher values of reactive astrocytes than the cell treated animals, indicating that the MNC hUCB cell treatment indeed effectively decreased astrocytic reactivity.

## Discussion

In the present study, we evaluated the effects of multiple MNC hUCB cell injections into a G93A SOD1 mouse model of ALS at different disease stages. The major findings in our study demonstrated that multiple MNC hUCB cell administrations into systemic circulation of G93A mice effectively: (1) delay disease progression; (2) improve animal survival; (3) enhance motor neuron survival; (4) modulate gliosis, and (5) reduce activation of microglia and astrocytes. Cell dose and the timing of initial treatment have a clear influence upon disease progression. Since the expression of mutant SOD1 gene (ΔCT) was similar for all G93A mice and there were no appreciable expression differences between groups, the benefits of MNC hUCB cells are noticeable. Administrations of 1×10^6^ cells initiated pre-symptomatically were most advantageous in both delaying disease onset and increasing lifespan whereas effective symptomatically-initiated cell infusions required higher cell doses (2.5×10^6^) to delay disease progression and so extend lifespan. Here, we are the first to demonstrate, from a translational viewpoint, treatment benefits when initiated at the symptomatic stage of disease. Thus, these results might provide a superior basis for the development of clinical trials, since the overwhelming proportion of ALS patients beginning treatment are already symptomatic.

Hence, cell-based therapy for ALS seems a more realistic and practical approach to developing neuroprotective strategies, protecting motor neurons and retarding disease progression [6]. Hematopoietic stem cells might provide these benefits. Previously, we have shown the effectiveness of human umbilical cord blood administration in a mouse model of ALS [45], [46]. Similar results have been noted by other researchers [48], [49]. Moreover, we have also demonstrated that a high single dose of 25×10^6^ MNC hUCB cells, injected intravenously into pre-symptomatic G93A mice, optimized survival and retarded disease progression [46]. However, to translate this experimental mouse therapy to the clinic would require impractically high cell doses.

The lessened effectiveness of MNC hUCB cells injected into symptomatic ALS mice had been expected, since motor neuron deterioration and neuroinflammation are already quite advanced when the initial symptoms manifest [50]–[53]. Since initiating treatment after symptoms appear is the most likely clinical scenario, high cell doses should be considered. However, smaller doses might be effective if intervention begins at the first appearance of disease symptoms. While ALS is a gradually progressive disease, treatment strategy by a series of cell infusions might be the best approach. The beneficial effect of multiple low-dose administrations of MNC hUCB cells has already been demonstrated in other models of neurodegenerative disorders: a transgenic mouse model of Alzheimer's disease [43] and a knockout mouse model of Sanfilippo syndrome type B [54]. Moreover, a recent report [55] indicated that serial intracerebroventrical injections of autologous cord blood-derived neural progenitors given to a child with global ischemic brain injury significantly improved neurological status.

In analyzing disease progression in G93A SOD1 mice, we consider rapid motor neuron degeneration to be a consequence of mutant SOD1 gene involvement. Therefore, we suggest that improved mouse survival after MNC hUCB infusions results from delayed disease progression. As expected, all groups of G93A animals suffered similar percentages of body weight and functional losses throughout their lives, due to the progressive nature of this disease. The differences between the groups continued to be the amount of time that elapsed to reach such endpoints. Body weight measurements closely followed the functional curves across time, since weight loss is a consequence of diminishing muscle mass and later muscle atrophy. By examining the functional status of each group through the Kaplan-Meier method, we were able to evaluate animal performance over time, without the bias of different survivals, a strategy already shown by Ohnishi et al. [56]. MNC hUCB cell infusions successfully improved most of the evaluated functions. Together, these data indicate that MNC hUCB cell treatment improves motor neuron survival, thus extending functional capabilities and mouse lifespan.

Although motor neuron death is a terminal event in ALS, directly associated with the clinical symptoms, disease pathogenesis involves multiple pathways in which neuroinflammation is a critical participant [9]–[11]. Reactive astrocytosis and activated microglia can be detected in cervical and lumbar spinal cord gray matter of G93A mice before disease onset [51], [52], progressively increasing until the end-stage of disease [50], [53]. The concept that non-neuronal cells contribute to the disease process, known as the “non-cell autonomous nature of motor neuron death”, is supported by several authors [57]–[59]. The role of activated microglia and reactive astrocytes as major inflammatory effectors contributing to motor neuron damage in ALS has been identified in various studies [9], [52], [60]–[65]. Astrocytes are directly involved in the establishment of motor neuron death, while activated microglia worsen local inflammation and disease progression [66], [67]. Therefore, inhibition of these inflammatory effectors in ALS could have a protective effect upon motor neurons.

In the present study, we determined significant reductions of astrocytosis and microglial density in the cell-treated G93A mice, indicating a modulatory effect of the MNC hUCB cells upon the inflammatory environment of the spinal cord. Previously, we demonstrated that a single injection of 25×10^6^ MNC hUCB cells into pre-symptomatic mice significantly decreases pro-inflammatory cytokines in the brain and spinal cord and reduces microglia density in the cervical/lumbar spinal cord [46]. We therefore suggest that the effect of multiple MNC hUCB cell administrations in decreasing neuroinflammation of the spinal cord, is neuroprotective and promotes motor neuron survival. Interestingly, although motor neuron densities are clearly distinct among the treatment groups, with better outcomes in mice receiving 2.5×10^6^ cells beginning at symptomatic stage (*Gr 1*) or 1×10^6^ cells at pre-symptomatically (*Gr 3*), such differences were not found in glial cell densities. Concerning astrocytes, it is possible that density evaluations are not as efficient as motor neuron counts in detecting subtle differences between groups. Regarding the microglia, however, the possibility remains that the cell treatment is successful not only in decreasing density, but also in promoting a shift from an “M1”, inflammatory, towards an “M2”, more tolerant immunological profile [10]. Since both microglia subtypes present similar phenotypes, the anti-Iba-1 antibody applied in the immunohistochemical evaluation would be insufficient to distinguish one from the other. Further studies are therefore necessary to clarify this point.

In the context of reducing neuroinflammation by repeated MNC hUCB cell administrations, we observed high concentrations of grafted human cells in the spleen, suggesting that this secondary lymphoid organ is acting as a reservoir of the administrated cells [68], [69]. Previously, we showed that even after a single small (1×10^6^ cells) intravenous injection of MNC hUCB into pre-symptomatic G93A mice, a majority of cells were identified in the white pulp of the spleen [45]. We hypothesize that in the spleen, the injected cells interact with the host cells and modulate the immunological response, lowering the inflammatory profile [70] and possibly protecting motor neurons in the remote spinal cord.

Numerous experimental studies have shown that the intravenous delivery of cells is effective in the treatment of ALS [45], [46], [48], [71], [72]. Moreover, it is considered a suitable route for translation into clinical application, due to its low invasiveness. In a previous study, we showed the migration of intravenously injected MNC hUCB cells and their differentiation into neural-like cells in the brain and spinal cord of G93A mice [45]. In the present study, we found that although the number of grafted human cells identified in the spinal cord was low and did not reflect the number of administered MNC hUCB cells, the treatment was effective. These results suggest that various factors secreted by the cells, rather than differentiation or cell-cell contact mechanisms, are the main therapeutic mediators. In fact, the same issue has already been approached in other neurological diseases such as stroke, in which functional improvement was disproportionally higher than the number of administered cells migrating to the site of injury [73]. Moreover, a recent report demonstrated that umbilical cord blood infusions improved neuromuscular transmission in G93A mice, indicating a direct effect of the treatment upon motor nerve function [71]. The authors consider that since the improvement was detected shortly after umbilical cord blood cell administration, cell replacement was a less probable mechanism of repair. In agreement with this idea, we consider that although cell replacement is possible and should not be overlooked, the results of the present study strongly support the actions of neuroprotection, which may include immunomodulation and secretion of trophic factors by the intravenously transplanted cells. Possibly, there is also some degree of motor neuron repair, which may be due more to endogenous pathways than administered cell differentiation.

In conclusion, we demonstrated that multiple injections of MNC hUCB cells are effective in improving motor neuron survival, likely due to decreasing macro- and microgliosis, and, in consequence, delaying disease progression and increasing lifespan of a mouse model of ALS. Beginning the cell injections pre-symptomatically provided the best outcome. Most important for translational purposes was proving the effectiveness of high cell doses initiated at the symptomatic disease stage. The present study results might provide essential information and strong impetus for future clinical trials.

## Materials and Methods

### Animals

All described procedures were approved by the Institutional Animal Care and Use Committee at USF, ID #R3416, and conducted in compliance with the *Guide for the Care and Use of Laboratory Animals*. One hundred and eight transgenic male B6SJL-TgN (SOD1-G93A) 1GUR mice (G93A; obtained from Jackson Laboratories, Bar Harbor, MA, USA), over-expressing human SOD1, carrying the Gly93→Ala mutation, were used. Before being assigned to the study, tail snips were obtained from each mouse and relative human SOD1 gene expression was quantified through standard procedure of real-time PCR [74], performed by Charles River Laboratories (Troy, NY, USA). The gene copy number was estimated through ΔCT, which is the difference in the threshold cycles between the transgene (human SOD1) and a mouse reference gene. The ΔCT is a direct index of the transgene copy number [75].

The G93A mice were randomly assigned to receive intravenously MNC hUCB cells or media weekly beginning at different disease stages. Injections were made into the penile vein of mice and started at pre-symptomatic (8–9 weeks of age) or symptomatic (13 weeks of age) disease stages and continued weekly until sacrifice. The cell dose of 1×10^6^ was injected into pre- and symptomatic mice and was chosen based on our previous study [45] showing effectiveness of this single cell dose in delaying disease onset. Repeated injections of 2.5×10^6^ cells were performed into symptomatic mice. This cell dose was chosen to deliver an optimal dose of 25×10^6^ cells, proven effective for both delaying disease onset and increasing lifespan of G93A mice [46], over a number of weeks. From a translational viewpoint, the weekly dose of 2.5×10^6^ cells injected into symptomatic mice should be roughly equivalent to 1–2 cord blood units administered to patients each month. The treated groups were divided as follows: *Group 1* – 2.5×10^6^ MNC hUCB (n = 27, symptomatic), *Group 2* – 1×10^6^ (n = 28, symptomatic), and *Group 3* - 1×10^6^ (n = 28, pre-symptomatic). There were two control groups: *Group 4* - G93A Media-injected (n = 25, symptomatic) and *Group 5* - transgenic mice (BL6/SJL) carrying the normal human allele for SOD1 gene (nSOD1, n = 20). All mice were maintained on a 12∶12 h dark∶ light cycle (lights on at 06:00 AM). Room temperature was 23°C. Food and water were available *ad libitum*. Upon progression of neurological symptoms, a highly palatable liquid nutritional supplement (Ensure Plus®) was placed on the cage floor, ensuring access by the animal.

### Preparation of MNC hUCB cells for transplantation

Cryopreserved MNC hUCB cells (U-CORD-CELL™, Saneron CCEL Therapeutics, Inc., Tampa, FL, USA) were thawed rapidly at 37°C then transferred slowly with a pipette into a centrifuge tube containing 10 ml of Dulbecco's Phosphate Buffered Saline 1× (DPBS), pH 7.4 (Mediatech, Inc., Manassas, VA, USA). The cells were centrifuged (400 rpm/15 min) at 12°C, the supernatant discarded and the process repeated. After the final wash, the viability of cells was assessed using the 0.4% trypan blue dye exclusion method before and following transplantation. Transplant cell concentrations were adjusted for each group: 25,000 cells/µl (2.5×10^6^ cells/100 µl/injection, *Group 1*) and 10,000 cells/µl (1×10^6^ cells/100 µl/injection, *Groups 2* and *3*).

### Cell or media administration

The MNC hUCB cells were delivered intravenously into the superficial dorsal penile vein of mice under anesthesia with Isofluorane (2–5% at 2 L O_2_/min) using a calibrated vaporizer-equipped induction chamber and nose cone. This route delivers the injected cells to the inferior vena cava and then to the right atrium for easy distribution throughout the body. Immediately prior to the injection, the penis was withdrawn from its protective sheath and wiped with 70% ethanol. A 31-gauge needle attached to a 100 µl Hamilton syringe was inserted through the skin at a 30° angle directly into the vein. During this procedure, the plunger was gently pulled back to aspirate blood back into the needle hub, ensuring that the needle was in the vein. Without moving the syringe, the plunger was then depressed to deliver the contents of the syringe into the penile vein. Once the cells (1×10^6^ or 2.5×10^6^) in 100 µl of vehicle (DPBS, pH 7.4) had been delivered, the needle was removed and pressure was applied to the vein for 30–60 seconds to prevent bleeding. The Media mouse group received 100 µl of DPBS, the same volume administered to the cell-transplanted mice. All animals, including controls, received cyclosporin A (CsA, 20 mg/kg per os) during the post-transplant period. Cell and media administrations were performed blind by independent investigators to avoid subjective bias.

### Characteristics of disease progression

The evaluation of animal disease progression has been previously described [45], [46]. All measures of disease progression were performed blind by independent investigators to avoid subjective bias. Body weight was assessed weekly throughout the study. Extension reflex, rotarod, and grip strength tests were started on week 9 and then repeated weekly thereafter. Five to six randomly selected cell-treated, non-treated, and control mice from each group were sacrificed at approximately 17 weeks of age, corresponding to 8 or 4 weeks after initial treatment at respectively pre-symptomatic or symptomatic disease stage, for immunohistochemical analysis of surviving motor neurons, glial cells and distribution of administered cells. The remaining G93A mice were behaviorally monitored until disease symptoms had progressed to the point of complete hindlimb paralysis, at which time the mice can no longer feed or care for themselves. After the last behavioral test, lifespan was determined.

#### Extension Reflex

The mouse was suspended by the tail and the extension of each hindlimb was observed. If the mouse showed normal hindlimb extension, a score of 2 was given. A score of 1 indicated partial hindlimb extension. If no extension was observed, the score was 0.

#### Rotarod

The mouse was placed on a 3.2 cm diameter axle rotating at a speed of 16 rpm (Omnitech Rotoscan, Omnitech Electronics, OH, USA). The latency (seconds) that the mouse stayed on the rotating axle during a 3 minute maximum period was counted.

#### Grip strength test (IDTECH-BIOSEB, France)

The mouse was held by the tail and carefully placed with all 4 paws on the grid. The animal was gently pulled by the tail and a sensor recorded muscle strength (Newtons) with which the mice resisted the pull. The test was performed three times during approximately one minute and the average of the tests was recorded.

### Tissue preparation

Mice (n = 5–6/group) at 17 weeks of age and the remaining mice from each group, when demonstrating an inability to move and reach food and/or water due to hindlimb paralysis and muscle atrophy, were sacrificed under deep chloral hydrate (10%) anesthesia and perfused transcardially with 0.1 M phosphate buffer (PB, pH 7.2) followed by 4% paraformaldehyde (PFA) in PB solution. The cervical/lumbar segments of the spinal cord and brain were removed, post-fixed in 4% PFA, and then cryoprotected in 20% sucrose in 0.1 M PB overnight. Coronal sections of the spinal cord and sagittal sections of the brain at 30 µm were cut in a cryostat, every fifth section was thaw-mounted onto slides, and the tissue was stored at −20°C for immunohistochemical analysis. The spleen, liver, kidneys and lungs were also removed, cut in a cryostat, and stored at −20°C for future analysis of administered cell distribution.

### Immunohistochemistry

#### Immunohistochemical staining of MNC hUCB cells

For identification of MNC hUCB cells in the cervical/lumbar spinal cords, brains, and organs (lungs, liver, spleen, and kidneys), serial tissue sections were stained with the human-specific marker (HuNu) as we described previously [45]. Briefly, the mouse monoclonal antibody (HuNu, 1∶50, Millipore, USA) was combined with the secondary antibody, monovalent goat anti-mouse Fab′ fragment conjugated to FITC (1∶200; Jackson ImmunoResearch, USA), and incubated at room temperature (RT) for 2 hours. The tissue sections on the slides were pre-incubated with 10% normal goat serum (NGS), 1% normal human serum and 0.3% Triton 100× in PBS, for 30 min at RT and, subsequently, incubated with the previously prepared antibody cocktail overnight at 4°C. Next day, the slides were thoroughly washed in PBS and coverslipped with Vectashield containing DAPI (Vector Laboratories, USA). The tissue was then examined under epifluorescence using an Olympus BX60 microscope.

#### Immunohistochemical staining of motor neurons in the spinal cord

Serial sections of the cervical and lumbar spinal cord were rinsed in PBS to remove the freezing medium. The tissue sections were pre-incubated in a blocking solution of 10% NGS and 3% Triton 100× in PBS for 60 min at RT, followed by overnight incubation with rabbit polyclonal anti-choline acetyltransferase primary antibody (1∶200, Abcam, USA) at 4°C. On the next day, the slides were rinsed in PBS and incubated with biotinylated goat-anti-rabbit secondary antibody (1∶300, Vector Laboratories, USA), 2% NGS, and 0.3% Triton 100× in PBS for 60 min at RT. After several rinses in PBS, an avidin-biotin-peroxidase enzyme complex (ABC-Elite kit, Vector, USA), followed by 3,3-diaminobenzidine chromatogen (DAB, Pierce, USA) were used to verify motor neurons within the spinal cords. Tissues were then dehydrated, coverslipped with synthetic resin mounting medium (Permount, Fisher-Scientific, USA) and examined under a bright field microscope (Olympus 60×).

#### Immunohistochemical staining of astrocytes and microglia in the spinal cord

Tissue preparation and pre-incubation followed the same procedures listed in the previous section (*immunohistochemical staining of motor neurons in the spinal cord*). For astrocyte staining, the tissues were incubated overnight with rabbit polyclonal anti-glial fibrillary acid protein primary antibody (GFAP, 1∶500, Abcam, USA) at 4°C. For microglial staining, tissues were incubated overnight at 4°C with rabbit polyclonal anti-ionized calcium binding adapter molecule-1 (Iba-1, 1∶2000, Wako, Japan). After primary antibody incubation, the slides were rinsed in PBS and incubated with biotinylated goat-anti-rabbit secondary antibody (1∶500, Vector Laboratories, USA), 2% NGS, and 0.3% Triton 100× in PBS for 60 min at RT. The tissue was then rinsed in PBS and incubated with avidin-biotin-peroxidase enzyme complex (ABC-Elite kit, Vector, USA), followed by 3,3-diaminobenzidine chromogen (DAB, Pierce, USA). Slides were then dehydrated, coverslipped with mounting medium and examined under a bright field microscope (Olympus 60×).

#### Motor neuron counts in the spinal cord

In preparation for stereological estimation of the motor neuron numbers in the ventral horn, an initial tissue section was randomly selected at one anatomical border of the spinal cord level (cervical or lumbar) to be examined. Thereafter, every fifth section of the spinal cord was used. Series of sections previously stained with anti-choline acetyltransferase (anti-ChAT) antibody were analyzed. The number of motor neurons was determined using unbiased, systematic stereological random sampling and the optical fractionator method [76] using the Stereo Investigator software package (MicroBrightfield, VT, USA). Spinal cord volume was measured by Cavalieri volume estimates, using the Stereo Investigator software. Outlines of the anatomical structures were done using a 10× objective and cell quantification was conducted using a 40× objective. Identification of motor neurons for counting was based on ChAT expression, cell size and shape. Motor neuron density is expressed as number of cells per mm^3^.

#### Astrocyte and microglial cell counts in the spinal cord

Analysis of astrocyte and microglial cell density in the cervical and lumbar spinal cords from 17 weeks old mice was performed using a computerized image analysis program (Image-Pro Plus, Media Cybernetics, Inc., Silver Springs, MD, USA) as we previously described [46]. Briefly, measurements of cervical/ lumbar ventral horn area were first performed by determining the cross-point of a line passing the central canal perpendicular to the midline. The area of ventral gray matter was determined below this line in the right and left cervical/ lumbar spinal cords in coronal sections (n = 6/slide) from each mouse group at predetermined uniform intervals (150 µm). Astrocytes or microglial cells were counted within right and left ventral gray matter of the cervical and lumbar spinal cords. Density of cells was determined as cell number per mm^2^.

For astrocytes, the proportion of reactive versus non-reactive cells was also determined, based on cell morphology. Reactive astrocytes present larger cell bodies and thicker, easily visible processes, as opposed to non-reactive cells, which have more delicate features.

#### Data analysis and statistical methods

Data are presented as means ± S.E.M. One-way ANOVA with Tukey's Multiple Comparison test was used to compare SOD1 gene expression and percentage of decline in body weight and functional tests between mouse groups. The same test was used to compare astrocyte, microglia and motor neuron densities and HuNu positive cell counts between mouse groups. The Kaplan-Meier method was used to determine differences in survival rates between groups of G93A mice with or without cell transplants, and is reported as a chi-squared value (χ2). For body weight and functional evaluations, an endpoint corresponding to the end-stage of disease was established, based on the mean percentage of decline of motor function or body weight in all G93A mice. The number of weeks elapsed for each animal to reach the established endpoint was determined and the resulting data were then evaluated through Kaplan-Meier method. For all analyses, p<0.05 was considered significant.
